# Validation of the GenoType^® ^MTBDR*plus *assay for detection of MDR-TB in a public health laboratory in Thailand

**DOI:** 10.1186/1471-2334-10-123

**Published:** 2010-05-20

**Authors:** Rapeepun Anek-vorapong, Chalinthorn Sinthuwattanawibool, Laura Jean Podewils, Kimberly McCarthy, Keerataya Ngamlert, Busakorn Promsarin, Jay K Varma

**Affiliations:** 1 Bangkok Metropolitan Administration, Health Laboratory Division, Bangkok, Thailand; 2 Thailand MOPH - U.S. CDC Collaboration, Nonthaburi, Thailand; 3 U.S. Centers for Disease Control and Prevention, Atlanta, USA; 4 Bangkok Metropolitan Administration, Department of Disease Control, Bangkok, Thailand

## Abstract

**Background:**

Over the past several years, new diagnostic techniques have been developed to allow for the rapid detection of multidrug resistant tuberculosis. The GenoType^® ^MTBDR*plus *test is a deoxyribonucleic acid (DNA) strip assay which uses polymerase chain reaction (PCR) and hybridization to detect genetic mutations in the genes that confer isoniazid (INH) and rifampn (RIF) resistance. This assay has demonstrated good performance and a rapid time to results, making this a promising tool to accelerate MDR-TB diagnosis and improve MDR-TB control. Validation of rapid tests for MDR-TB detection in different settings is needed to ensure acceptable performance, particularly in Asia, which has the largest number of MDR-TB cases in the world but only one previous report, in Vietnam, about the performance of the GenoType^® ^MDR*plus *assay. Thailand is ranked 18^th ^of 22 "high-burden" TB countries in the world, and there is evidence to suggest that rates of MDR-TB are increasing in Thailand. We compared the performance of the GenoType^® ^MTBDR*plus *assay to Mycobacterial Growth Indicator Tube for Antimycobacterial Susceptibility Testing (MGIT AST) for detection INH resistance, RIF resistance, and MDR-TB in stored acid-fast bacilli (AFB)-positive sputum specimens and isolates at a Public TB laboratory in Bangkok, Thailand.

**Methods:**

50 stored isolates and 164 stored AFB-positive sputum specimens were tested using both the MGIT AST and the GenoType^® ^MTBDR*plus *assay.

**Results:**

The GenoType^® ^MTBDR*plus *assay had a sensitivity of 95.3%, 100%, and 94.4% for INH resistance, RIF resistance, and MDR-TB, respectively. The difference in sensitivity between sputum specimens (93%) and isolates (100%) for INH resistance was not statistically significant (p = 0.08). Specificity was 100% for all resistance patterns and for both specimens and isolates. The laboratory processing time was a median of 25 days for MGIT AST and 5 days for the GenoType^® ^MTBDR*plus *(p < 0.01).

**Conclusion:**

The GenoType^® ^MTBDR*plus *assay has been validated as a rapid and reliable first-line diagnostic test on AFB-positive sputum or MTB isolates for INH resistance, RIF resistance, and MDR-TB in Bangkok, Thailand. Further studies are needed to evaluate its impact on treatment outcome and the feasibility and cost associated with widespread implementation.

## Background

Drug-resistant tuberculosis (TB) has emerged as an important global public health threat. The World Health Organization (WHO) estimates that 489,000 cases of multi-drug resistant TB (MDR-TB), defined as infection with a *Mycobacterium tuberculosis *(MTB) strain resistant to at least isoniazid (INH) and rifampin (RIF), occur annually, predominantly in Eastern Europe and Asia [1]. Treatment involves prolonged use of "second-line" anti-TB drugs that are less effective, less tolerated, more toxic, and more expensive than "first-line" anti-TB medications [2]. Under optimum program conditions, cure rates for drug-susceptible TB exceed 90%; for MDR-TB, cure rates infrequently exceed 70% [3]. In most high-burden TB countries, MDR-TB is only diagnosed after prolonged treatment with first-line TB drugs and clinical recognition that treatment has failed. Treatment of drug-resistant TB with standard first-line drugs, instead of a regimen designed according to the resistance pattern, has several potential adverse consequences: patients remain on inadequate treatment longer, increasing the risk of treatment failure or death; regimens inadequate to kill MTB amplify resistance to drugs to which their isolates were previously susceptible; and patients remain infectious, promoting transmission to close contacts [4]. Because of this problem, the WHO is recommending that countries immediately expand their capacity for culture-based drug-susceptibility testing (DST) and consider new, molecular-based assays for diagnosing drug resistance [5,6].

The internationally accepted gold standard for MDR-TB diagnosis is demonstration of MTB growth in cultures inoculated with INH and RIF. Even using modern broth-based culture systems, obtaining DST results from sputum specimens still takes several weeks [7]. New assays have been developed to detect resistance faster using genotype, rather than phenotype. The GenoType^® ^MTBDR*plus *test is a deoxyribonucleic acid (DNA) strip assay which uses polymerase chain reaction (PCR) and hybridization to detect mutations in the *inhA, katG*, and *rpoB *genes that confer INH and RIF resistance [8]. A recent meta-analysis found that the GenoType^® ^MTBDR*plus *assay and one other similar commercial test have a pooled sensitivity of 98% for detecting RIF resistance and 89% for detecting INH resistance [9]. Specificity averages 99% for RIF and INH [9]. Testing can be performed on isolates or AFB-positive sputum specimens and can return results in 8 hours, making this a promising tool to accelerate MDR-TB diagnosis and improve MDR-TB control.

Although the GenoType^® ^MTBDR*plus *assay has been studied in several laboratories, there is wide variation in circulating MTB strains across the globe [10,11], and false negative results occur due to unique genetic mutations [9,12-17]. Validation in different settings is needed to ensure acceptable performance, particularly in Asia, which has the largest number of MDR-TB cases in the world but only one previous report, in Vietnam, about the performance of the GenoType^® ^MDR*plus *assay [18]. Thailand is 18^th ^on the list of 22 "high-burden" TB countries in the world, with over 90,000 cases occurring annually [19]. A national survey in 2002 found that the rate of MDR-TB was 1% in previously untreated TB patients and 20% in previously treated patients; a second survey in 2006 found that the rate of MDR-TB had increased to 1.7% in previously untreated patients and 34.5% in previously treated patients [20]. We have previously validated the feasibility and performance of broth-based culture and DST at the Bangkok city public TB laboratory in Thailand [7]. In this study, we compared the performance of the GenoType^® ^MTBDR*plus *to a broth-based DST assay for detecting INH resistance, RIF resistance, and MDR-TB in AFB-positive sputum specimens and isolates in the same laboratory.

## Methods

### Setting

Between July and September 2008, we evaluated the performance characteristics of the GenoType^® ^MTBDR*plus *assay at the Bangkok Metropolitan Administration (BMA) Health Laboratory Division, the primary clinical laboratory for the city's TB control program. Before study implementation, we tested 50 MTB isolates of known resistance patterns from WHO's External Quality Assurance program to evaluate laboratory proficiency in using this assay. After this, the performance characteristics of the GenoType^® ^MTBDR*plus *assay were assessed in two populations: (a) 50 stored MTB isolates with known resistance patterns to INH and/or RIF from pulmonary TB patients in Bangkok; and (b) 164 stored acid fast bacilli (AFB)-positive sputum specimens that were submitted to the BMA laboratory by 163 TB patients for routine culture and DST. All MGIT AST results were blinded to the microbiologists performing the GenoType^® ^MTBDR*plus *assay.

### Routine testing of sputum specimens

Sputum specimens were processed using the U.S. Centers for Disease Control and Prevention (US CDC) recommended method of N-acetyl-L-cysteine 4% NaOH-2.9% citrate (final concentration of NaOH 1%). Following incubation at room temperature for 15 minutes, specimens were concentrated, decanted, re-suspended, and then examined for the presence of AFB using Ziehl-Neelsen (ZN) method. The remaining suspension was used to inoculate two Lowenstein-Jensen (LJ) tubes and one Mycobacterial Growth Indicator Tube (MGIT, Becton-Dickinson). Residual processed sputum was frozen at -70°C and used for GenoType^® ^MTBDR*plus *testing. MGIT cultures were incubated in the MGIT BACTEC 960 for six weeks. All cultures flagged as positive were removed, examined for AFB using ZN staining, and subcultured to LJ and confirmed as MTB using classical biochemical tests including niacin accumulation, nitrate reduction, and inhibition to para-nitrobenzoic (PNB) acid [20]. All isolates identified as MTB underwent MGIT AST for INH, RIF, streptomycin, and ethambutol, using previously described methods [7]. All positive MGIT vials were stored at room temperature for the duration of the assessment to allow discrepant testing.

### DNA isolation from clinical specimens and MTB isolates

AFB positive sputum specimens graded as scanty, 1+, 2+ and 3+ were prepared for the GenoType^® ^MTBDR*plus *assay by first concentrating 500 μL of the residual processed specimen in a microcentrifuge (10,000 × g, 15 minutes, room temperature). The supernatants were decanted and the pellet was re-suspended in 100 μL of distilled water, then inactivated by incubating the bacteria in a heating block for 20 minutes at 95°C. Cells were sonicated in an ultrasonic bath for 15 minutes, and concentrated for an additional 5 minutes. The supernatants were transferred to a new tube to be stored for PCR.

Isolates were prepared for the GenoType^® ^MTBDR*plus *assay by first sub-culturing stored clinical strains to broth culture then to LJ. We then used approximately 1 loop full of colonies taken from the LJ to prepare a bacterial suspension in 300 μL of distilled water. This suspension was inactivated, sonicated and concentrated using the same procedures applied to sputum specimens.

### DNA amplification

Amplification was performed by combining 35 μL of primer nucleotide mix (PNM) with 5 μL of 10× PCR buffer (containing 15 mM MgCl_2_), 2 μL MgCl_2 _(25 mM MgCl_2_), 3 μL molecular grade H_2_O, 0.2 μL (1 unit) HotStar *Taq *polymerase (QIAGEN, Hilden, Germany), and 5 μL of the bacterial suspension for a total final volume of 50.2 μL. The amplification profile for direct patient material as described by the manufacturer was used for all bacterial suspensions. First, the template DNA was denatured for 15 minutes at 95°C, followed by 10 cycles consisting of 30 s at 95°C and 2 minutes at 58°C, with an additional 30 cycles consisting of 25 s at 95°C, 40 s at 53°C and 40 s at 70°C. The final cycle consisted of an 8 minute run at 70°C.

### Hybridization

Hybridization was performed manually using a shaking water bath/Twincubator^® ^preheated to 45°C. Twenty microliters of denaturation solution were mixed thoroughly in a plastic 12-well tray with 20 μL of amplified sample and incubated at room temperature for 5 minutes. One milliliter of hybridization buffer was added to each well and mixed. We then placed 1 pre-labeled test strip into each well, and incubated the test strips and solutions for 30 minutes at 45°C. All solutions were completely aspirated following incubation. One milliliter of stringent wash solution was then added to each strip and incubated for 15 minutes at 45°C. Once all solutions were completely aspirated, we applied 1 mL of rinse solution to each strip for 1 minute. The rinse solution was then completely removed and 1 mL of diluted conjugate was added to each strip, and incubated for 30 minutes. After incubation, all solutions were removed, and the test strips were rinsed twice by using a rinse solution for 1 minute, followed by distilled water for 1 minute. All solutions were completely aspirated between rinses. We then added 1 mL of diluted substrate to each strip and incubated the test strips protected from light for up to 20 minutes. All solutions were removed, and the reaction was stopped by rinsing twice with distilled water. The test strips were allowed to dry, and then taped to the GenoType^® ^MTBDR*plus *assay worksheet for interpretation.

### Repeat testing and discrepant analysis

Sputum specimens and isolates resulting in inconsistent development of bands on the MTBDR*plus *strip, and/or no MTB control band underwent repeat PCR and hybridization from the extracted DNA. Isolates with discrepancies between the susceptibility results of the GenoType^® ^MTBDR*plus *assay and MGIT AST were sent to US CDC for sequencing.

### Statistical analyses

The time from positive culture in the laboratory to the time DST results were read was calculated using the GenoType^® ^MTBDR*plus *assay and the MGIT AST method for each specimen. All statistical tests were performed using Stata 10.0 (College Station, TX). Statistical significance was established at an alpha level of 0.05.

### Ethical review

This project underwent formal ethical review at the U.S. CDC and Bangkok Metropolitan Administration. It was approved as a public health program evaluation, not requiring individual informed consent.

## Results

Phenotypic testing identified INH resistance in 14/50 (28%) isolates and in 29/164 (18%) sputum specimens, and RIF resistance in 6/50 (12%) isolates and in 19/164 (12%) sputum specimens. The GenoType^® ^MTBDR*plus *assay had a sensitivity of 95.3% (41/43), 100% (25/25), and 94.4% (17/18) for INH-resistance, RIF-resistance, and MDR-TB, respectively (Tables 1a and 1b). Of the 41 specimens that were identified as INH-resistant using the GenoType^® ^MTBDR*plus *assay, 32 had a mutation in the *katG *gene, 6 in the *inhA *gene, and 3 in both genes. For INH resistance, the sensitivity was lower for sputum specimens (93%) than for isolates (100%), but the difference was not statistically significant (p = 0.08). Specificity was 100% for all resistance patterns and for both specimens and isolates. We were able to obtain interpretable sequence data for one of two sputum specimens with INH resistance detected by conventional testing but not molecular testing; this isolate was identified as wild type.

**Table 1:** Performance characteristics of the GenoType^® ^MTBDR*plus *assay.


**A. Performance characteristics of the GenoType^® ^MTBDR*plus *assay for the detection of RIF- and INH-resistance in comparison to drug-susceptibility test results using the MGIT BACTEC 960.**									

		**INH Resistance**				**RIF Resistance**			
		**MGIT AST Result**				**MGIT AST Result**			
**Specimen Type**	**GenoType^® ^MTBDR*plus *Result**	**+**	**-**	**Sensitivity** **(%)**	**Specificity (%)**	**+**	**-**	**Sensitivity (%)**	**Specificity (%)**

**AFB+ Sputum**	**+ (Resistant)**	27	0	93.1	100	19	0	100	100
	**- (Sensitive)**	2	135			0	145		

**Stored Isolate**	**+**	14	0	100	100	6	0	100	100
	**-**	0	36			0	44		

**All Specimens**	**+**	41	0	95.3	100	25	0	100	100
	**-**	2	171			0	189		

**B. Performance characteristics of the GenoType^® ^MTBDR*plus *assay for the detection of MDR-TB in comparison to drug-susceptibility test results using the MGIT BACTEC 960.**									

	**MDR-TB** **MGIT AST Result**								

**Specimen Type**	**GenoType^® ^MTBDR*plus *Result**	**+**	**-**	**Sensitivity** **(%)**	**Specificity (%)**				

**AFB+ Sputum**	**+ (Resistant)**	12	0	92.3	100				
	**- (Sensitive)**	1	151						

**Stored Isolate**	**+**	5	0	100	100				
	**-**	0	45						

**All Specimens**	**+**	17	0	94.4	100				
	**-**	1	196						

+ = Resistant; - = Sensitive

For the 211 specimens with dates recorded for time from the culture result to the DST result, the laboratory processing time for the GenoType^® ^MTBDR*plus *assay was significantly shorter than MGIT AST: 5 days compared with 25 days (p < 0.01) (Table 2). The most marked advantage in laboratory processing time was evident when specimens were processed directly from AFB-positive sputum smears (3 days vs. 24.5 days; p < 0.01).

**Table 2:** Comparison of laboratory processing times using the GenoType^® ^MTBDR*plus *assay and the MGIT BACTEC 960 for drug-susceptibility testing.


	**MGIT BACTEC 960**			**GenoType^® ^MTBDR*plus***	
**Specimen Type**	**Culture Result to Identification** **median days (IQR)**	**DST Initiation to DST Result** **median days (IQR)**	**Total Time from Culture to DST** **median days (IQR)**	**DNA Extraction to DST Result** **median days (IQR)**	**Total Time from Culture Result to DST** **median days (IQR)**

Direct Sputum +	15 (12 - 19)	9 (7 - 11)	24.5 (21 - 29)	3 (2 - 5)	N/A

Stored Isolate	14 (12.5 - 19.5)	9 (7 - 12)	25 (21 - 28.5)	2 (1.5 - 2)	16 (14 - 21)

All Local Specimens	15 (12 - 19)	9 (7 - 11)	25 (21 - 29)	2 (1 - 3)	5 (3 - 9)*

IQR = interquartile range (25^th ^and 75^th ^percentile)

* Pooled for all specimens: for direct sputum AFB+ time from DNA extraction to DST result; for isolates time from culture result to DST result.

## Discussion

In Bangkok, the GenoType^® ^MTBDR*plus *detected INH resistance, RIF resistance, and MDR-TB with a high sensitivity and 100% specificity in both isolates and AFB-positive sputum specimens, with substantial reductions in turn-around time compared with MGIT AST.

This study provides assurance that TB patients in Thailand with MDR-TB detected with the GenoType^® ^MTBDR*plus *assay should immediately commence treatment with second-line anti-TB drugs. The 100% specificity corresponds to a positive likelihood ratio of near infinity, suggesting that, regardless of the pre-test odds of MDR-TB, the post-test odds from a positive test are sufficiently high to warrant MDR-TB treatment. Ideally, the GenoType^® ^MTBDR*plus *assay should be performed on AFB-positive sputum specimens, rather than isolates, to take advantage of the more than three week acceleration in turn-around-time. Use of the assay on AFB-positive sputum and rapid commencement of MDR-TB treatment for patients with genotypic resistance has the potential to improve patient outcomes, reduce TB transmission, and reduce amplification of resistance to first-line drugs (e.g., ethambutol, pyrazinamide, and streptomycin) to which the isolate may not yet be resistant.

One weakness of the GenoType^® ^MTBDR*plus *is the lack of 100% sensitivity for MDR-TB, attributable to the test's failure to detect all mutations that confer INH resistance. Two AFB-positive sputum specimens were confirmed as INH resistant by MGIT AST and sensitive by the GenoType^® ^MTBDR*plus*. We were able to obtain interpretable sequence data of the promoter region for *inhA *and *katG *for one specimen which confirmed the result of the GenoType^® ^MTBDR*plus*. INH resistance in this strain may be due to mutations in regions other than codon 315 of *katG *or nucleic acid positions -15, -16 and -8 in the *inhA *promoter region; or mutations in genes not represented on the test strip, such as *ahpC-oxyR*, and *ndh *[21,22]. However, a recent evaluation of 160 INH resistant isolates in Thailand found that 92.5% (148) of the gene mutations were found in codon 315 of *katG *and the *inhA *promoter and coding regions (127 and 22, respectively) [23]. These findings support the utility of the GenoType^® ^MTBDR*plus *assay for detecting the majority of genetic mutations conferring INH resistance in Thailand. As we were not able to obtain quality sequencing results for our second specimen, the reason for the discordance remains unclear. Advances in understanding the molecular epidemiology of INH resistance may contribute to improved test performance.

Our validation study was conducted using stored sputum specimens and isolates from TB patients in Thailand. Therefore, our turn around time using the GenoType^® ^MTBDR*plus *for identification of RIF and INH resistance in AFB positive sputum specimens is based on the time from confirmed AFB positive smear results to interpretation of the GenoType^® ^MTBDR*plus*. The turn around time for isolates is based on the time required to subculture strains of MTB, obtain growth, perform and interpret results of the GenoType^® ^MTBDR*plus *assay. On average, results were available using GenoType^® ^MTBDR*plus *on AFB-positive sputum specimens in 3 days and in 16 days for isolates; compared with 25 days using MGIT AST. Our laboratory uses the manual method for the GenoType^® ^MTBDR*plus *assay and performs DNA extraction and PCR on the first day and hybridization on the second day. Interpretation of the test strips required an additional 1-2 days. Laboratories with adequate staff may reduce their turn around time by performing DNA extraction, PCR, and hybridization in one day, and by using the automated system (GT-Blot 48; Hain LifeScience GmbH, Germany). Turn around time from specimen collection to susceptibility result will be determined during the implementation phase of our study.

This study evaluated a large number of sputum specimens at a routine public health facility in a high-burden TB setting. Most evaluations of the GenoType^® ^MTBDR*plus *assay have involved a relatively small number of specimens (usually less than 50) and were conducted in primarily academic facilities located in low TB burden, high income countries [9,12-17]. The only other evaluation that involved a large number of samples from a public health facility in a high-burden TB country was done in South Africa [16,17]. Test performance in 536 sputum specimens was similar to our findings, although the test showed the additional ability to detect MTB and drug-resistant MTB in smear-negative, culture-positive specimens. The major limitation of our study is that it involved a relatively small number of sputum specimens compared with the number routinely processed in the laboratory, which may have been an insufficient sample to detect differences in test performance between isolates and AFB-positive sputum.

In conclusion, the GenoType^® ^MTBDR*plus *assay has been validated as a rapid and reliable first-line diagnostic test on AFB-positive sputum or MTB isolates for INH resistance, RIF resistance, and MDR-TB in Bangkok, Thailand. Whether it should be implemented into routine public health programmatic use, however, is not yet clear. Further studies are needed to evaluate its impact on treatment outcome and the feasibility and cost associated with widespread implementation.

## Competing interests

The authors declare that they have no competing interests.

## Authors' contributions

RA, KN, and BP performed the laboratory experiments. CS and KM provided oversight and technical assistance for all laboratory procedures, and CS was responsible for collecting, validating, and managing the laboratory database. CS, LJP, KM and JKV developed the research design and protocol, and LJP and JKV served as the principal investigators for the study. RA, CS, LJP, KM, KN, BP, and JKV analyzed and interpreted data, and LJP, KM, and JKV drafted the manuscript. All authors have contributed to, seen, and approved the final, submitted version of the manuscript.

## Pre-publication history

The pre-publication history for this paper can be accessed here:

http://www.biomedcentral.com/1471-2334/10/123/prepub
